# Savoring the service: A qualitative study of Latine community health workers’ health promotion efforts

**DOI:** 10.1371/journal.pgph.0006654

**Published:** 2026-08-12

**Authors:** Elayne Zhou, Daniela Arcos, Lyric N. Russo, Violet A. Flores, Jacob Aguilera, Marco Maldonado, Stanley J. Huey, Jessica L. Borelli

**Affiliations:** 1 Department of Psychology, University of Southern California, Los Angeles, California, United States of America; 2 Department of Psychological Science, University of California, Irvine, Irvine, California, United States of America; Johns Hopkins University Bloomberg School of Public Health, UNITED STATES OF AMERICA

## Abstract

Latine communities have long faced stark health inequities and systemic barriers to accessing healthcare services. Community health workers (CHWs) serve as a key link between patients and the often-daunting healthcare system, providing crucial health education and support. Despite this important yet demanding role, little is known about CHWs’ personal experiences of community health promotion and how they might draw upon their work for strength and endurance. The present study explored the potential for relational savoring, a procedure that elicits feelings of closeness and connection, to capture CHWs’ experiences of community health promotion. CHWs (*n* = 30) from California community health agencies completed audio-recorded tasks in which they recalled and mindfully savored service provision memories. Transcripts were examined via inductive thematic analysis. Four themes were identified: Fulfilling Experiences, Sources of Resilience, Challenges of Community Health Work, and Support Net. The present study expands our understanding of important caregiving processes in community health workers. Findings may inform future efforts to improve individual and group-level mental health outcomes in Latine communities.

## Introduction

Community health workers (CHWs) in low-income Latine communities embody a striking paradox: as integral members of this underserved group, they are uniquely equipped with the cultural understanding and intimate knowledge to foster conditions for optimal community health. However, their vulnerability to poor health outcomes stems from a dual burden: they often face many of the same challenges as their clients, and these challenges are further exacerbated by the demands of intensive service responsibilities [1]. Despite growing interest in and research on CHWs and their effectiveness in promoting the health of others, CHWs’ own well-being has been largely neglected [2,3]. Additionally, what is currently known about how to promote healthcare workers’ resilience has been driven by research on other healthcare providers, such as physicians and nurses [4,5]. This limited research focus restricts the development of effective interventions that could be specifically tailored to support CHWs. Guided by a strengths-based approach, the present study delves into CHWs narratives of community care provision, which may provide insight into how to help insulate CHWs from the wear and tear of their demanding roles. This study aims to advance our ability to promote improvements in health outcomes at both individual and community levels by illuminating the experiences of key members of underserved Latine communities.

### Addressing Latine community health needs

CHWs support the health initiatives and overall well-being of Latine communities by connecting various components of the community health system and ensuring its effectiveness. The healthcare needs of Latine communities are indisputable. The California Health Care Foundation reports that Latine people are overrepresented among residents living below the federal poverty line, and are also more likely to report no usual source of healthcare, delayed care due to cost or lack of health insurance, and more difficulty finding doctors than non-Latine white Californians [6]. It is no surprise, then, that Latine Californians report poorer health than other racial-ethnic groups [7]. In response to these diverse health challenges, CHWs have mobilized to deliver targeted interventions for a range of chronic health conditions in Latine communities including initiatives from the Centers for Disease Control and Prevention [8]. CHWs have also been instrumental in the successful recruitment and retention of Latine populations in research studies to facilitate the development of more culturally responsive measures and interventions [9–11]. Though support for the effectiveness of CHW delivered interventions on improving health behavior and outcomes is mixed [12–14], various studies have demonstrated greater improvements in client outcomes compared to alternative interventions [15].

The adaptability and flexibility of CHWs in responding to the varying needs of community members allows for a personalized approach to address these challenges effectively. However, capturing the resultant wide-ranging experiences of CHWs engaging with the community they serve, and indeed even characterizing CHWs themselves, becomes increasingly complex. The groundbreaking *Community Health Worker National Workforce Study* defines CHWs as community members that work, either paid or unpaid, with the local healthcare system and that share important lived experiences and identities with the groups they serve [16]*.* The state of California employs almost 15% (8,940) of the national estimate of 61,300 employed CHWs [17]. The median income for CHWs in California stands at $50,310 compared to the national median income for CHWs, $46,190 [17]. According to the National CHW Survey [18], the majority (83%) of CHWs in the U.S. were employed full-time, with the remaining 17% working on a part-time, volunteer, or per diem basis, or compensated via honorarium. CHWs are also employed across a variety of settings (e.g., hospitals, community health clinics, government, social assistance, education, residential care, and administration and support) [19]. Relatedly, criteria for recruitment can vary significantly, for example, provision of services through Medi-Cal may dictate requirements, or if the CHW is employed within a state that requires CHW certification for employment. Globally, there is even more variation, with CHWs in Ghana required to complete 40 hours of training for volunteer positions, while some CHWs in Nigeria complete roughly 3 years of training for minimum-wage positions [20]. Still, there is still much that is unknown about the nature of CHW work, some of which may be difficult to capture with quantitative methods.

There is also a lack of consensus surrounding CHW’s roles and specific responsibilities, in part because of the incredible heterogeneity of CHWs. In fact, the National CHW Survey (2021) found that CHWs used at least 97 different professional titles to describe their roles and responsibilities. Typically, the terms “community health worker” and “promotoras [de salud]” are most commonly used, and done so interchangeably, which may obscure important nuances in the essential community-engaged work they carry out. Importantly, promotores are distinct subset of CHWs, community members who leverage their existing professional expertise and resources to advocate for the betterment of their own community. While promotores are invaluable and have received a deserved amount of interest from researchers and policy makers alike, they only constitute a portion of the dedicated individuals embedded in community health systems who form the spectrum of CHWs more generally. All CHWs actively serve the community through different means, making them vital to the sustainability and smooth functioning of community health systems.

An over-emphasis on front-line CHWs who are directly delivering health education and promotion interventions inadvertently overlooks the broader range of individuals that interface with the community at large to create conditions for health and well-being. Additionally, focusing primarily on intervention delivery and outcomes may inadvertently apply an incongruent medical model of health on CHWs who operate from a social model of health [21,22]. Said differently, rather than a singular focus on the effects of modern medicine on health outcomes (medical model), CHWs examine and address various social determinants such as social, cultural, political, and other environmental factors that facilitate optimal health (social model, [23]). The present study seeks to widen the lens through which CHWs are examined and understand their experiences more comprehensively. By doing so, we can broaden our conceptualization of the diverse contributions of CHWs and develop strategies that support their individual well-being as well as their capacity to care for the community at large.

### Challenges faced by community health workers

Amidst the COVID-19 pandemic, CHWs have experienced heightened burnout and psychological distress, grappling with unique stressors and demands that set them apart from other essential frontline health workers [1,24,25]. Burnout is defined as a psychological phenomenon resultant from chronic interpersonal stress within the workplace, bringing about unmanageable exhaustion, cynicism and depersonalization, and a decreased sense of self-efficacy [26]. Predictors of burnout can be both individual (e.g., existing mental and physical health risks) and organizational (e.g., caseload, work-life balance, organizational culture and support), while social support may serve to buffer healthcare providers from work-related burnout [27]. Similarly, long-term burnout has been linked to negative impacts on individual health, personal relationships, and professional functioning [28]. The negative health impacts of job-related burnout on mental well-being in essential frontline workers, particularly during the pandemic, are well-documented [29,30], yet CHWs have received limited attention.

Prior to the pandemic, CHWs and the community health system were already overstretched, the state of which was exacerbated with the devastation caused by COVID-19. Within both the U.S. as well as other countries, various studies documented increased risk for burnout, traumatic stress, occupational overwhelm, and vulnerability [31–33]. In qualitative studies, CHWs further specified the various challenges that emerged in their work: work-life balance, gender power imbalance with male clients, emotional load, limited English fluency, difficulty working with healthcare providers, managing conflict between clients’ cultural beliefs and the community health agendas, and lack of transportation [34]. The pandemic has also worsened preexisting financial and psychosocial challenges faced by CHWs, and the growing demand for support from their clients has further encroached upon an already precarious work-life balance [1]. These same studies show that, despite these difficulties, CHWs have embraced their expanded roles in the pandemic response and utilized the internal and organizational resources available to them.

Though suggestions to address burnout in healthcare workers in the context of COVID-19 have mostly excluded CHWs, these recommendations, such as interpersonal connections and self-reflective practices, present a promising foundation for next steps [35]. By examining the distinctive bond between CHWs and the communities they serve, the present study will shed light on strategies for mitigating poor health outcomes and enhancing the overall well-being of these essential workers.

### Resilience in community health workers

Considering the demanding nature of healthcare, particularly within underfunded systems responding to a global health crisis, it is crucial to investigate how CHWs safeguard their well-being and mitigate the risks of burnout and resultant adverse health outcomes. Resilience, which involves adaptability, responsiveness, and strength in the face of challenges or demands [36], may be a particularly important target for healthcare workers. A recent integrative review identified community and interpersonal connections including moral purpose and duty, connections, collaboration, and organizational culture as key factors of resilience in hospital healthcare workers in the pandemic [37]. Concerningly, additional studies found that resilience in nurses decreased from pre-pandemic levels [38]. Yet, what is currently known about resilience in healthcare workers is often limited by who is considered a “healthcare professional.” While healthcare workers in hospitals and community health agencies may share goals and face similar challenges in promoting overall health, CHWs occupy a markedly different position in terms of both their duties and the specific settings they work in. Gaining insights into resilience among various types of healthcare professionals serves as an initial albeit insufficient step towards understanding the experiences of CHWs, who have been otherwise neglected. Given that resources mobilized to improve well-being among healthcare professionals have overlooked CHWs, very little is known about how CHWs may be best supported. In a recent qualitative study conducted by Marquez and colleagues [1] to identify the impacts of the COVID-19 pandemic on promotoras/es, various themes were identified, including social disconnection, increased community needs, expanded service duties, and the emotional challenges of representing a “community in pain.” Still, promotoras/es additionally reported rewarding experiences and a shared sense of “emotional unity.” These initial findings hint at the ways in which promotoras/es may leverage their service experiences to navigate an unprecedented and evolving landscape of care provision, though this focus does not include other kinds of CHWs. The present study will explicitly focus CHWs’ narratives around what factors are important in bolstering and supporting their well-being in underfunded healthcare systems.

### Savoring, social connection, and enhanced positive experiences

*Relational savoring* (RS), a procedure through which positive interpersonal experiences are deliberately appreciated [39], may be a helpful tool in revealing CHWs’ conceptualizations of social connection in their work. RS is an emotion regulation strategy related to general *savoring*, which involves attending to, appreciating, and even enhancing positive experiences in one’s life [40]. With RS, the individual savors specific experiences of felt or provided support and security within relationships [41]. For example, a person could savor a time when someone said something supportive to them before they embarked upon a frightening challenge, or a time when they themselves comforted someone who was going through a difficult life event.

RS can also be delivered in a guided intervention format. When delivered in this way, RS protocols consist of a five-step reflective process that aims to promote the mindful enjoyment of a particular relational experience: 1) sensory reflection, 2) emotion reflection, 3) meaning making, 4) future-oriented reflection, and 5) internalizing the savoring exercise as a whole [41]. RS intervention studies have reported outcomes such as improved positive emotion, relationship quality, cardiovascular reactivity, and even adherence to COVID-19 regulations [39,42–44]. The primary objective of RS is to cultivate connection and facilitate flourishing, a state of optimal psychological well-being associated with resilience in the face of adversity [45]. Given its attachment focus, to date, RS has only been administered in the context of individual relationships (e.g., mothers savoring experiences with their children, individuals in long-distance romantic relationships savoring experiences with their partners). Thus, applying the concept of RS to CHWs’ relationships with a community, the focus of the current investigation, is novel and can help shape future directions. In the present study, RS will serve as a lens through which we will examine the thought processes of CHWs around service experiences. Narratives generated from the RS process can provide valuable insights into the salient experiences of CHWs and how they make sense of their community service encounters during challenging times.

### Present study

By solely focusing on CHWs’ roles as facilitators of change in their communities without considering the nuance and potential stressors inherent in their work, we risk overlooking crucial factors that may impact both their own well-being and their ability to promote community health. Therefore, the present study aimed to understand not only how CHWs engage with the communities they serve but also how these interactions impact their own resilience in the face of adversity. This work will advance our understanding of CHWs’ experiences of community health promotion and help identify ways to support them more effectively.

The study sought to explore two aims:

1. *To use RS to investigate CHWs’ engagement with the community and identify salient service experiences that are particularly impactful.*
2. *To characterize how CHWs derive value and meaning from service experiences (i.e., why are these salient experiences impactful for CHWs?).*

## Materials and methods

### Participants

Participants included in the study (*N* = 30) were current employees of community health agencies serving low-income Latine families. Note that our decision to use the term “Latine” in describing the present study was collaboratively decided with our community health agency partner. We use the term “Latine” in this manuscript due to the fact that it is additionally inclusive of gender diverse individuals and people living in Latin America above and beyond the more historically utilized term “Hispanic.” Still, we do not assert that the term “Latine” is all-encompassing or representative of the incredibly unique and diverse experiences and individuals categorized into this grouping. To be eligible, participants needed to be at least 18 years of age and work as a community service provider at a community service agency. There was no eligibility criteria based on specific job responsibilities (e.g., community health related administrative, outreach, planning, program or intervention delivery) or other sociodemographic factors. Study participation was completed in either English or Spanish depending on participant preference.

### Ethics statement

Study approval was granted through the University of California, Irvine Institutional Review Board under approval number #1596. Formal, written informed consent was obtained.

### Recruitment

Participants were drawn from a larger, ongoing multi-site study [46]. CHWs were recruited between 10/1/2022–11/30/2023. Data was accessed for research purposes on May 1st, 2023, and authors did not have access to identifying information during analysis. Recruitment was carried out via in person methods such as distributing flyers and delivering presentations through community agencies. Printed and digital flyers and brochures were distributed through community health agencies. Our primary source of recruitment was the Latino Health Access (LHA), a community health agency serving primarily Latine communities in the Santa Ana area in California. LHA engages in disease prevention and intervention through the provision of culturally relevant care and education to reach particularly underserved individuals. Interested participants who contacted our lab completed a brief phone screening process, and if eligible, were scheduled for a virtual study appointment during which they provided consent. The majority of the sample (*n* = 22) completed study participation in Spanish, with the remaining participants completing study procedures in English.

All participants were California residents (see Table 1 for participant sociodemographic characteristics), identified as Latine/Hispanic, and were largely of Mexican origin (*n* = 20), one participant listed their country of origin as Venezuela, and the remaining participants (*n* = 9) did not report country of origin. Participants’ average age was 45.23 years old (*SD* = 11.94 years) and the average number of years participants had been working as a community service provider was 9.33 years (*SD* = 7.82 years). The majority of participants were born outside of the U.S. (73.3%), female (96.7%), and had completed 12 years of education (76.7%). No participants reported education beyond 12 years. Primary job duty reported was community outreach (56.7%). The median household income was $40,000 - $44,999, compared to the state median household income of $84,097 [47]. Still, our sample was above the 2023 poverty line for a 4-person household in California ($30,000) [48]; the average Latine/Hispanic household is about 3.8 individuals [49].

**Table 1:** Sociodemographic characteristics of participants.

Characteristic	*n*	%
Gender		
Female	29	96.7
Male	1	3.3
U.S. Born		
Yes	8	26.7
No	22	73.3
Marital status		
Single	8	26.7
Separated	2	6.7
Divorced	5	16.7
Widowed	1	3.3
Married	11	36.7
Cohabitating	3	10.0
With children	22	73.3
Annual Family Income		
Less than $5,000	1	3.3
$15,000 - $19,999	1	3.3
$20,000 - $24,999	1	3.3
$25,000 - $29,999	1	3.3
$30,000 - $34,999	3	10.0
$35,000 - $39,999	4	13.3
$40,000 - $44,999	6	20.0
$45,000 - $49,999	2	6.7
$50,000 - $54,999	3	10.0
$55,000 - $59,999	2	6.7
$60,000 - $69,999	1	3.3
$70,000 - $74,999	1	3.3
$80,000 - $84,999	1	3.3
$85,000 - $89,999	1	3.3
$100,000 - $149,999	2	6.7
Highest educational level		
Less than 12 years	7	23.3
12 years	23	76.7
Community Health Employment		
Volunteer	2	6.7
Employed	13	43.3
Missing	15	50.0
Hours Worked		
Part Time	7	23.3
Full Time	8	26.7
Missing	15	50.0
Primary Job Duties		
Planning	4	13.3
Community outreach	17	56.7
Program delivery	2	6.7
Administrative work	3	10.0
Other	4	13.3

### Procedures

The parent study (NCT05560893; registered September 15th, 2022) utilizes a randomized controlled waitlist design, wherein all participants are randomized into either an experimental group or a waitlist group following a baseline assessment. Participation in the parent study occurs for a total of 5 hours across 7 time points for the experimental group; CHWs completed a baseline assessment, weekly intervention sessions for four weeks, a post-intervention follow up assessment, and a 3-month follow up assessment.

Participants, regardless of condition assignment, were included in the present study if they had completed both the initial baseline assessment and the first intervention session. During the baseline assessment, participants completed a demographic survey via Qualtrics. The following week, they completed a 30-minute RS task over Zoom with research assistant interveners. Participants were compensated $55 for their participation via Venmo, physical gift card, or a virtual gift card.

### Measures

#### Demographics.

At the baseline visit, participants reported on relevant sociodemographic characteristics (previously reported in Table 1). A separate attempt was made to further collect details on employment and hours. Of the 15 participants who responded, the majority (86.67%) were employed by their community health agency and worked full-time hours (53.33%).

#### Savoring.

Participants completed a two-part relational savoring task with research assistant interveners (see S1 Appendix for the protocol). The RS protocol was adapted for use with CHWs from a script originally developed for use with caregivers ([41,44] for a detailed description of the original protocol and selections from the original script).

In part 1, interveners engaged participants in a brief mindfulness exercise to set the tone for RS; this step is not included in the analysis. Interveners then guided CHWs through memory selection, with the goal of eliciting a memory of a time during which the participant acted as a secure base (i.e., source of reliability and/or security) or safe haven (i.e., source of safety and/or comfort) either for the community at large or for a specific community member. Interveners were instructed specifically to help select a salient memory that did not evoke negative emotions. In the event that the participant was unable to produce a memory with explicit secure base or safe haven content, interveners were trained to highlight aspects of the support that the participant provided to the community or community member that positioned the participant as a safe haven or secure base.

In part 2 of the RS task (memory reflection), the participant was prompted to recall the memory and savor various sensations, cognitions, and emotions tied to the salient experience over the course of 10 minutes. Participants were instructed to verbalize their experience throughout the guided reflection. During this portion of the RS task, interveners prompted participants five times to attend to different features of the recalled experience. First, participants recalled details of the memory. Next, they recalled emotions experienced and corresponding physical sensations, and were asked to reexperience those sensations. Then, interveners highlighted the secure base/safe haven role and encouraged participants to reflect on this ongoing relationship with their community. Participants were then asked to practice a future focus, extrapolating this experience to future engagement with the community. Finally, participants mindfully reflected on the event in a non-directed manner and were encouraged to verbalize their reflections.

## Data analytic plan

### Background

To address the study aims, we conducted a secondary data analysis of qualitative data from savoring sessions. Specifically, we employed *reflexive thematic analysis* (TA), to extract, investigate, and describe patterns and narratives from their data. Given the dearth of research on CHWs’ service experiences and well-being, we utilized an *inductive* (data-driven) and atheoretical approach [50]. An inductive and atheoretical approach is additionally valuable when researchers do not belong to the community being studied.

We additionally employed several methods to establish *trustworthiness* in order to maximize accountability, replicability, and rigor [51]. Trustworthiness can be assessed via the criteria of credibility, transferability, dependability, and confirmability. Lincoln & Guba [52]) operationalized *credibility* through member checking, testing the alignment between researchers’ interpretations of the data with the participants’ own experiences. In the present study, we incorporated a two-stage member-checking process, described in more detail in phases 5 and 6 of the analytic process below; we presented findings to non-coding research team members as well as to the participants themselves via a focus group to elicit feedback to inform interpretation and presentation of findings. *Transferability* is the quality of generalizability via inclusivity of information so future researchers can determine how findings may transfer [51]. Our coding team incorporated transferability by including, where possible, context-rich quotes with additional information (i.e., “thick description”) in quote excerpts to assist future researchers in determining how findings might transfer to other samples or environments. *Dependability* requires an auditable, well-documented, and easily followed process [53]. For the current study, we prioritized dependentability by incorporating memos where possible that explained decision making or coder perspectives, and documenting the process of consensus coding and resolving disagreements in coding. Once the previous criteria are met, *confirmability* is then achieved; the process through which findings are reached can be understood [52].

Braun & Clarke’s approach to reflexive TA includes six phases: *familiarization*, *coding*, *generating initial themes*, *reviewing and developing themes*, *defining and naming themes*, and *writing up*. To ensure faithfulness to the participants’ experiences, we aimed to capture an essentialist or realist account of the data to “report experiences, meanings and the reality of participants” [54]. Similarly, we identified themes at a *semantic or explicit* level, wherein participant accounts and patterns are described and later interpreted as they are written.

### Phase 1: Data familiarization

Savoring sessions were transcribed using Trint, which uses artificial intelligence to automatically transcribe audio content to text [55]. Trint is compliant with the European Union’s General Data Protection Regulation directive. All data are stored on Amazon Web Services and encrypted at-rest. Original recordings are automatically deleted within 30 days of upload on Trint. Research assistants then reviewed transcriptions for accuracy and ensured the removal of any identifying information. A four-person coding team, bilingual in both English and Spanish, was assembled. Coders identified their ethnic background as the following: Latino or Mexican-American (coder 1), Latino or Mexican immigrant (coder 2), Latina or Ecuadorian (coder 3), and Colombian and Mexican (coder 4).

At the beginning of Phase 1, the lead author provided an overview of qualitative research, planned phases of TA, and an introduction to the analysis software. All phases were documented for auditability and were conducted in English. We used Dedoose (Version 9.0.17), a user-friendly qualitative data analysis software, to collaboratively analyze and process transcriptions (*Dedoose software*; [56]). In this phase, all coders reviewed every transcript and engaged in *reflexive journaling*. Coders added memos to each excerpt to document their real-time reactions to the data, assumptions and/or biases, and their thinking process to inform coding. Coders were instructed to read actively and aim to answer the following questions: 1) How does this participant make sense of their experiences? 2) What assumptions do they make in interpreting their experience? and 3) What kind of world is revealed through their accounts? [57].

At the end of this phase, the coding team met as a group to consolidate memos and collectively process initial reactions to the data, with the goal of producing “ideas about what is in the data and what is interesting about them” [54, p. 88].

### Phase 2: Initial code generation

During Phase 2, codes were assigned to the data. Braun & Clarke define codes as the ‘building blocks of analysis’ where a single word or concise phrase is identified and applied to a data segment of interest to the research question(s) [57]. At the beginning of this phase, each coder produced some preliminary ideas around a possible code list. Coders were provided general instructions on how to approach the data and what constitutes a code. After creating a tentative code list and accompanying definitions (i.e., what the code is and is not), the coding team independently coded three transcripts. After initial coding, the template was revised to refine coding categories and definitions in cases of significant overlap or lack of clarity. Transcripts that were coded with the preliminary code list were re-coded with the final code list.

During weekly meetings, the coding team engaged in consensus coding [58] to resolve discrepancies on transcripts coded independently, reviewing disagreement as a group and collectively determining the final code for each excerpt. Consensus coding or having multiple coders review each transcript increases the credibility of the analysis. Together, consensus coding and peer debriefing serve as forms of *researcher triangulation* where multiple perspectives help minimize individual bias and promote a more comprehensive understanding of the data [52]. Consensus coding was particularly necessary and appropriate for the present analysis and coding team due to the makeup of the team—that is, two of the four coders were interveners delivering the RS protocol (Coder 1 had administered RS to 27% of participants and Coder 3–23% of participants). All consensus coding processes and decisions were documented.

### Phase 3: Initial theme generation

After all relevant data excerpts were coded, codes were examined and organized into themes. Themes are broadly and flexibly defined as “captur[ing] something important about the data in relation to the research question, and represent[ing] some level of *patterned* response or meaning within the data set” [54, p. 82]. The coding team was trained on the process of arranging codes into themes before embarking on this phase.

Using the Dedoose qualitative charts, the coding team reviewed a word cloud of codes applied, a code frequency matrix (S1 Fig), a code presence matrix (S2 Fig), and a code co-occurrence matrix (S3 Fig). The coding team created and workshopped a list of themes and related definitions. To assist in visualizing how different codes cluster together or relate to others, a preliminary thematic map was created through consensus. This phase ended with all codes initially organized into themes. The coding team also began brainstorming with the first author around how themes fit together in the broader narrative.

### Phase 4: Review of themes

The coding team next engaged in a refining process by: 1) checking individual themes against coded data excerpts and 2) examining how well the themes fit in with the dataset as a whole [57]. Importantly, the team ensured that themes were both internally homogeneous and externally heterogenous [59]. To that point, it is important to acknowledge that one code (Gratitude from Clients) was deemed conceptually relevant to two themes and thus was sorted into both Fulfilling Experiences and Sources of Resilience. Through weekly meetings, the thematic map created in the previous stage was revised and finalized to reflect any combined or further segmented themes. Any decisions made that affected the hierarchy of themes and assignment of codes were documented.

### Phase 5: Defining and naming themes

In this penultimate phase, a final table was created of theme names, definitions, and representative data excerpts (translated from Spanish to English if needed). The thematic map was then presented to other research assistants, graduate students, and staff that were familiar with the data but did not serve as coders for additional member checking. Feedback on the order and presentation of the themes was implemented.

At the end of this phase, study participants were invited to attend a 1.5 hour focus group at LHA, of which a subset (*n* = 13) chose to attend. During this meeting, the first and second author presented on the research findings and engaged participants in a discussion to elicit feedback, in both Spanish and English. Three questions were posed: What do you think of these themes? Are you in agreement with our learnings? and Are there any changes that you would make to the themes? Participants in this focus group were compensated $40 and provided a meal for their time. Submitting results to participants for feedback allowed the research team to do a final quality check [60].

### Phase 6: Generating the final report

In the last phase, all findings and logical processes were synthesized into the present report. For a final time, the team engaged in peer debriefing through a presentation to non-coding members of the research team. Lastly, the report was reviewed by a qualitative and mixed methods expert and a committee of scholars, and a Spanish summary was generated for ease of sharing with participants and community health agencies (S2 Appendix).

## Results

**Fig 1:**
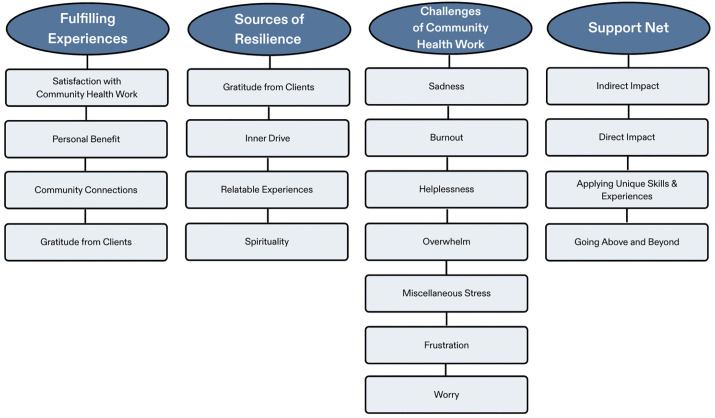
Thematic map displaying themes and respective codes from relational savoring transcripts.

All excerpts included in the analysis reached consensus during the coding process. Codes were sorted into a total of four themes: *Fulfilling Experiences*, *Sources of Resilience*, *Challenges of Community Health Work*, and *Support Net* (see Fig 1 for thematic map).

S3 Appendix displays representative data extracts or quotes within each theme; Spanish quotes were translated to English for the purposes of the present report. The results of the thematic analysis are presented below in response to each of the research questions posed, translated from Spanish to English as needed.

### (RQ1a) How do community health workers engage with the community?

CHWs form a support net for their clients, providing a web of resources for their community. Much of this support is achieved through acting as a reliable source by meeting clients’ needs. For instance, CHWs provide funds to assist clients facing eviction, host a regular community food bank, provide emotional support for clients, and connect clients to necessary healthcare or legal services. CHWs described witnessing the fruits of their labor and the immediate impact of their efforts on their clients’ lives and wellbeing (*direct impact)*.

“Everything was like good for me, it was good like that, very gratifying, because I felt good, useful because you helped a lot of people… But gratifying because you helped a lot of people with–with food and with this financial help. There was–there was a–there was a help. And–and they helped each other with their clients so that they could open, so that they could eat. It went well and I liked it. I felt very useful at that moment.”

Several specifically noted the importance of cultivating independence and self-reliance in their clients so that they may help themselves in the future (*indirect impact)*.

“And this is for me more than me helping fiscally-that is, directly to a person, for me I feel that I am not helping but leaving something planted mhm right? And something sown is that that person can be independent and not have to come all the time to the promoter [Name] or the promoter [Name] or whoever to be able to resolve their, their issues…”

Many CHWs also expressed a desire to “plant a seed” by empowering their clients to help others in the community, and in essence, compounding their own impact by positioning their clients as agents of further change. In fact, several CHWs recalled seeing their former clients joining the community health “support net” themselves by volunteering or sharing resources with others in need. One CHW described the value of acknowledging impact no matter how big or small, describing it as a “granito de arena” or grain of sand. Regardless of the type of services provided, CHWs often applied their own unique skills and experiences developed externally to their work to find creative solutions to community needs (*applying unique skills and experiences)*.

“I feel responsible that if I have, for example, my abilities to make crafts I feel responsible that I have to teach people...and I feel I feel happy that I can teach others my abilities, and that those people can then teach [others].”

For example, CHWs incorporated their artistic abilities, listening skills, or community organizing in the services they provided.

Perhaps most striking was the CHWs’ recounting of experiences where they improved the lives of their clients above and beyond their job description or the immediate presenting need—that is, working beyond their scheduled hours, sustaining long-term friendships with clients, and supporting community members in a manner that exceeded professional or formal expectations (*going above and beyond)*. A particularly illustrative example is the below quote translated from Spanish to English wherein a CHW, invested in the long-term success and well-being of both her client and the client’s family, proactively enrolled her client’s husband in English classes, and continued to not only provide encouragement but also keep up to date with his progress.

“Ah uh, since she speaks pure English, the one we are in, the one I put in school was her husband…I sent him because he didn’t want to and I told him, ‘Yes, look you have to do it’ and he said, ‘no’ that he will not want to do it. I told him, ‘No, yes, you are going to do it because I am telling you to.’ And so I enrolled him, I sent him and I am very proud because he is very consistent in his classes. He is on time to class, does his homework, I mean, he is taking it like really, really, something like really valuable to him.”

### (RQ1b) What service experiences are particularly salient and impactful?

As part of the RS intervention, CHWs were invited to mindfully recall positive, meaningful moments as part of their work. As such, it is no surprise that most commonly, CHWs spoke about the benefits and positive effects that they experienced as a result of their roles in the community. However, community health work is not without its challenges. Despite the focus on positive moments of connection in RS, CHWs raised various struggles resulting from the demands of their position–highlighting the emotionally demanding nature of community work, often performed by women.

CHWs frequently described, with emotion, the sense of fulfillment they derived from engaging with their community clients. CHWs described satisfaction with what they may achieve through their unique role (i.e., their [potential] contributions to the community at large)—that is, for some, the position they hold contributes to a realization of purpose or a meaning-making process (*satisfaction with community health work)*.

“Well, it is–it is– personally I do have that satisfaction of-of saying or maybe I have helped a bit, but I have contributed a bit to my community and also that I have realized that- for example, our community is a community that needs a lot of help… I think I can contribute so that the resources arrive to them, for them to take advantage of the resources that exist, because when I say that there are resources for us to take advantage of and for these to be utilized by those who really need it, right? That to me–me. Well, yes, it creates a lot of satisfaction and at the same time like suddenly to live, I think.”

Additionally, several CHWs described satisfaction with the work environment and their team, specifically describing collective efforts with coworkers and community members to make a lasting impact on their broader community through programming and advocacy. Relatedly, one of the most prevalent meaningful experiences described by CHWs was the sense of felt connection that they shared with community members via trust building, providing CHWs a strong feeling of belonging as well as the opportunity to provide a safe space for clients (*community connections)*.

“Mmm, well generally uh we feel very supported. The truth is, in this country, in this city, I am the only one from my family with my son, so practically they are my family, all my memories are with them.”

CHWs also noted other personal benefits that they perceived resulting from the position, including monetary support, though for some the financial incentive was a bonus to the broader fulfillment they experienced. Often, a particularly salient benefit that CHWs received from their clients was in the form of gratitude (*gratitude from clients)*. The majority of CHWs described moving displays of appreciation that they received from their clients, whether verbal or physical, which served as tangible reminders of the impact that they had made through their work.

“[My client said] ‘I attended the [event] and thanks to the [services] that you did with us, you gave me the desire to live, I changed, I changed my way of thinking.’ And she thanked me very much, so that left me totally impacted like, there are others memories, right, of other projects that I’m also involved in, [smacks lips] but this was the one that left me the most impacted, and of– of satisfaction and knowing that perhaps those two hours that we met a week we were working on–on her without realizing it and to change that way of thinking...”

Efforts were often mutually beneficial and CHWs described not only growing professionally through the lessons learned through working with the community but also receiving support themselves in times of need (*personal benefit)*. For example, in the quote below translated from Spanish to English, a CHW describes the bidirectional caregiving and carereceiving they have engaged in with their community clients.

“I think that it is super good because every person that comes into my life impacts me in some way, but at the same time I impact their lives. It’s like-like a mutual learning [process] and the support is mutual, because when I lost my mom, they [referring to community members served] were with me, they brought me flowers, umm they cooked for me so that I wouldn’t have to cook. Umm, when they evicted me they would tell me, ‘This is your house, don’t worry. Come here to the–to our house’s yard we will set up a tent. You are not alone.’”

Still, challenging or negative experiences with community health work were also present in CHWs narratives. CHWs empathized with the suffering, pain and loss experienced by community members, particularly during the pandemic (*sadness, stress)*.

“If they got sick, they died because sometimes they arrived at the clinics very, the people, very unwell, very unwell, I mean with those fevers and almost passing out and [they] would arrive at the clinics infected people and a lot of people. Even though it was painful because a lot of people started to lose their family members.”

Moreover, CHWs described feeling emotional and occupational fatigue (*burnout)*, wherein they expressed exhaustion, dissatisfaction with work responsibilities, and experiencing devaluation of their work, such as the below example translated from Spanish to English.

“To the point where we can put our limits ourselves, because we have to take care of our integrity, right? We cannot risk our lives for other people. And even more if [they] don’t want to receive help. So yeah…until now I don’t see that-that we play an important role or that-that our job is totally recognized because it is not recognized.”

Some CHWs expressed a sense of lack of control or helplessness in being able to fully resolve clients’ problems due to insufficient resources or funding (*helplessness)*.

“We can’t control who’s gonna be getting what money or if they run out of funds, who’s gonna help them. It’s kinda getting less rewarding now uh to help people because… I have no control.”

Similarly, CHWs shared a feeling of being overwhelmed due to the discrepancy between supply and demand (*overwhelm)*. A few CHWs expressed some nonspecific stress associated with community health work and the challenge of serving a struggling community while also themselves struggling with unmet needs, such as the below example translated from Spanish to English.

“Sometimes it’s a little, we can talk with a lot of people to help [them] but really what [you] are doing is how we feel at the same time we are helping and sometimes we ask ourselves mhm ‘how do I feel?’ Because these people don’t ask me ‘how do I feel?’ Maybe I have problems, I have to bring out [my] joy to the people so they don’t see me sad, nor frustrated, or sometimes I have depression problems but I have to face the community because the community too, well [they] go through worse things than my self.”

Some CHWs expressed frustration with and anger around systemic barriers or challenges that the broader community was facing (*frustration)*.

“So, I think there’s an element of criminalizing poverty…Instead of saying “who broke the rule and how do we punish them,.”..let’s see what happened, let’s see what resources we can offer that will benefit both parties.”

Additionally, several CHWs demonstrated a lack of self-efficacy or appeared preoccupied about their ability to meet their clients’ needs (*worry)*.

“Sometimes, you know, when you feel like, okay, you’re maybe not doing enough for someone and you kind of feel like I couldn’t help them maybe mhm….Like, I just there was no help for this person. So I had to just leave them in the middle of mhm their situation.”

Still, many of the challenges faced by CHWs appeared to stem from ongoing larger structural issues which actively complicate and impede their ability to meet community needs, which I would like to intentionally emphasize.

### (RQ2) How do community health workers derive value and meaning from service experiences?

Expressions of appreciation and gratitude from clients, while a source of fulfillment, also appeared to contribute to CHWs resilience and emotional strength (*gratitude from clients)*.

“Well yes I felt happy like no, no, I don’t want to cry because it tickled my stomach of happiness and of surprise. And it’s me and happiness. Happiness that someone came to my door and brought me flowers…I was all happy and with everyone that would want to talk to me I would tell them ‘this happened to me’ and that day I would tell them the story, but yes I go [feeling] well, I liked it.”

Community connections are intertwined with CHWs’ longevity and motivation. Many CHWs reported a sense of recognition of or identification with their clients’ experiences; some explicitly described memorable clients as reminders of their past selves or of a loved one, reflecting on shared needs and struggles as an impetus for action (*relatable experiences)*.

“Well, this is a success for us, because we were there at some moment in our lives, and I think that is the success of promotoras. The ‘oh shoot, me too, I can act and help.’ And you do it with all your heart. Of course. It’s the experience, then, that you understand the interconnectedness because of the [shared] experience…”

Indeed, several CHWs expressed intrinsic desires or an inner drive to help others, with some describing a past history of helping or volunteerism prior to working at their current agency (*inner drive)*.

“Well you see, in that moment my goal was to fulfill the need of the lady, which was the vaccine, mhm mhm, so I focused on that to see in what way I could help her, how I could help her, because I did not want to let her down, right right, I didn’t want her to feel like–like–feel insecure with me and later I be like ‘nothing can be done’, no no no, I was very focused in being able to satisfy her need.”

Finally, a number of CHWs made references to their religious or spiritual faith, specifically in terms of relying on and trusting in their faith to guide them through difficult decisions or challenging times, as well as considering the work and opportunity to aid their community as God-given (*spirituality)*.

“...I always try to always give thanks to God because I say ‘Yes, wow, I don’t know how it happened, but I did it and thanks to God that gave me the opportunity to do it’, right? Because I always thought of saying ‘Father, I have this and that type of responsibility that I carry, but without your help I will not be able to do it’ and I’m praying that everything goes well.”

### Focus group findings

In addition to the qualitative data we analyzed from savoring sessions, we also received important feedback regarding the implications of our findings, and future research directions from the focus group we conducted with participants as part of our process of rigor. We present them here with our general findings, though it is important to note that our methodology was different due to the aim of the focus group (i.e., member checking); general comments from the focus group discussion and recommendations are presented here, though the focus group and specific quotes were not recorded.

Though the present study explicitly adopted a strengths-based perspective, CHWs emphasized that they did not want a focus on resilience to water down their yet unmet needs, particularly with regards to systemic and structural barriers. Specifically, CHWs called for a “de-normalization of the way [we] live” regarding unfair compensation and challenging living situations. One CHW stated “de buena gente no vives” or that simply being a “good person” is insufficient to survive and thrive. Rather, CHWs urgently need greater mental health support as well as the legitimization of their expertise via their role as CHWs. Specifically, they described that, despite “pouring their souls” into their demanding roles, the lack of recognition and dignification of their lived and work experiences served as a barrier to employment. Yet, like many other CHWs in California [61], CHWs participating in the focus group strongly opposed efforts by the state to move towards certification of CHWs, stating that certification will become a further barrier for CHWs’ own work and employability. Notably, CHWs made explicit that their calls for fair compensation and recognition of their experience also extend to research partnerships and the growing interest from institutions in CHWs.

## Discussion

The current study employed a qualitative approach to investigate CHWs’ experiences of serving low-income Latine community clients, and how various experiences may be differentially related to CHWs’ own well-being. Importantly, the study focuses on CHWs’ individual and collective experiences rather than considering CHWs as a means to an end (i.e., effectiveness in intervention delivery and promoting community health outcomes). Employing RS as a tool to understand how CHWs engage with their client community and make meaning from these experiences, we identified four qualitative themes (ordered here by thematic “thickness” or most to least represented): Fulfilling Experiences, Support Net, Sources of Resilience, and Challenges of Community Health Work. Findings bolster our understanding of community caregiving processes and their role in the lives of CHWs, further illuminating how community health work may be linked to CHWs’ well-being and work-related longevity.

Our study was the first to utilize RS as a method of uncovering the intricate and dynamic relationships between CHWs and the communities that they serve; however, several of our findings complement the minimal existing literature on CHWs. Findings indicate that 1) CHWs engage with the community by comprising a support net for a community in need, 2) CHWs are particularly impacted by both fulfilling (e.g., personal benefits via professional development, tangible impact, and community connections) and challenging experiences (e.g., sadness, emotional burnout, and overwhelm) in their work, and 3) CHWs derive strength and resilience from various intrinsic (e.g., inner motivation, religiosity) and extrinsic sources (e.g., shared or relatable experiences) in striving to benefit their client community.

Themes identified in the current study align closely with similar studies in the U.S. and complement the global literature on CHWs. Past qualitative work with promotoras/es in the U.S. reported similar findings around professional transformation, socio-emotional and organizational challenges, and commitment to community health work by way of close interpersonal relationships [34,62]. Qualitative research conducted in rural India on a subgroup of CHWs, Accredited Social Health Activists, similarly highlight how personal traits and experiences enhance community health work, frustration with structural limitations and disempowerment, and the importance of financial incentive in service delivery [63]. The current study contributes an added layer of understanding of community health work, as past research on CHWs has to our knowledge exclusively centered the experiences of promotoras/es, who are typically primarily engaged in program delivery, but are not representative of all individuals who work as CHWs to improve community well-being.

### Recommendations

Through both RS as a way to share their narratives around community health promotion and feedback via a focus group, CHWs illustrated several of their existing strengths as well as areas for further support. First, relationships are paramount to successful community health work. CHWs find both internal (e.g., within the organization) and external relationships (e.g., with clients and collaborators) to be rewarding and beneficial. Unsurprisingly, when interpersonal and organizational support is lacking, then CHWs find it difficult to engage in the work and meet community needs. While CHWs form a support net for others, they themselves require emotional and financial support systems. These findings have important implications for programming and structure within community health agencies. As such, we join in calls for fair pay, protected employment, and increased financial compensation for CHWs befitting the invaluable experience and unique perspective that they bring to their work. Our findings complement national reporting that more than half of CHWs do not endorse receipt of a living wage, and the majority do not receive additional compensation for overtime work [18]. On a global scale, compensation structures vary drastically but studies point to inadequate compensation and additional challenges around regularity of payments that directly impede community health work and contribute to turnover such as in sub-Saharan African countries and rural India [63,64]. A further point of contention in CHW pay is in performance-based incentives, wherein one qualitative study in rural India found that CHWs may be more encouraged to prioritize medical based services or targets over other important facets of social health [65]. Regardless, across low- and middle-income countries, unreliable or insufficient pay threatens the sustainability of CHW programs; further, there is evidence that fair pay does not decrease intrinsic motivation, as some may argue, but rather that it may enhance CHWs’ ability to carry out community-driven work [66].

Community health agencies should also consider the benefit of introducing peer support models for all CHWs, regardless of their primary job duty or volunteer status, and protecting such emotional and social support as part of grant and funding applications. The literature on similar peer support programming conducted one-on-one, in group settings, and online for healthcare providers shows promise as a supportive service that may allow providers to circumvent stigma associated with professional help seeking behavior, and foster positive working environments [67]. Relatedly, national reports reveal that approximately one third of CHWs do not feel that their workplace supports their self-care and mental health [18]. Programs such as RS that extend CHWs experience of meaningful and rewarding experiences and thus, possibly the existing benefits of the work may also warrant further consideration [46].

Moreover, CHWs draw from various sources of resilience; thus, different aspects of community health work are salient to different CHWs. Taking a personalized approach to fostering resilience in CHWs from the beginning of their work with communities may contribute to their longevity in and satisfaction with the role. Identifying the sources of motivation for each CHW (e.g., spirituality, interpersonal connections, values around contributing to or helping their community) and regularly creating space for CHWs to deliberately access and experience those values may also increase their motivation or job satisfaction in the long term.

It is important, too, that CHWs perceive that their work is making a tangible impact in their immediate and broader community. Agencies are encouraged to regularly acknowledge the accomplishments of individual CHWs and as a group—specifically, agencies may want to emphasize how the contributions of each CHW, regardless of the division of their time and labor, further the grander mission of the social model of health in the community. Beyond the individual, work recognition and respect of CHWs’ expertise on a structural level may look like increased inclusion of CHWs in programmatic leadership and organizational decision-making; on a national scale, 61% of CHWs report being able to shape their roles and career [18]. In sum, our takeaways in analyzing CHWs’ experience of community health promotion illustrate the importance of proactively investing in CHWs well-being.

### Limitations and future directions

While the present study expands our understanding of how CHWs not only serve but also draw strength from their client community, several study limitations should also be considered. First, though a sample size of 30 is comparable to typical sample sizes in qualitative research [68], it remains limited in its generalizability. Specifically, only one participant identified as male, which means our results may not represent varied CHW experiences. This is particularly true given gendered perceptions of the role that contribute to the underrepresentation of men in the CHW workforce and their implications on belongingness of male CHWs [69]. However, the low percentage of male CHW participation in our study aligns with state-wide gender distributions and global estimates that approximately 70% of CHWs are women [66,70]. Additionally, our sample of CHWs were situated in California, where as of the 2020 Census, Latine or Hispanic identifying individuals make up the largest racial or ethnic group in the state, which may not represent the experience of CHWs in other states. Further, CHW experiences may differ depending on the demographic makeup of their client community, resources of their community agency and neighborhood served, and the policies impacting different areas. In addition to the limited sample size, we did not evaluate differences in responses based on factors that could be important to evaluate, including hours worked (e.g., full-time, part time), work status (volunteer, paid), or legal status, due to the structure of our data collection, the sensitivity of the questions and the preferences of our community partners, which could affect the conclusions drawn and would be important to explore in future research.

Second, the present study is a secondary data analysis of qualitative data and thus did not utilize a qualitative interview guide intentionally devised to answer the specific research questions of the current study. However, RS is a useful tool by which researchers were able to gain insight into the close relationships and relationship processes that CHWs share with their client community and tap into important personal and community narratives. Still, the present study took a number of measures to meet suggested standards for conducting secondary data analysis of qualitative data regarding clarity (i.e., between the present and parent study) rigor (e.g., trustworthiness via documentation, peer debriefing, member checking, and including research team members from the parent study), and delineating limitations [71]. Third, two members of the coding team had served as interveners in the parent study, delivering the RS protocol to 50% of the participants included in the study, which may have introduced bias into the coding process. While including research members from the parent study is actually recommended in secondary data analysis of qualitative data [71], precautionary steps were taken to reduce coder bias. To address this concern, the decision was made to conduct coding via consensus between all coders rather than to divide coding and conduct inter-rater reliability analyses. Peer debriefing and member checking processes also served to address coder bias. Still, potential coder bias should be considered in the interpretation of the study findings.

## Conclusions

While future research should be guided by research findings, it is equally if not more important to also ensure research is responsive to community needs.

Though the results highlight important factors relevant to individual resilience and work-related longevity, we would be remiss to paint individual resilience as a solution to structural and systemic challenges that hinder CHWs. In their efforts to promote community health, policymakers must incorporate CHWs perspectives in co-creating inclusive and sustainable policies with wide-reaching public health implications, lest they unintentionally further health inequities by adopting policies (e.g., certification processes and requirements) that exclude CHWs, creating further hoops to jump through for already overextended and underserved essential workers. Additionally, policymakers should consider policies that may benefit other healthcare workers but overlook lay CHWs. For example, despite healthcare worker burnout being named a priority for the U.S. Surgeon General in a recent advisory calling for more fair pay, and equitable and empowering practices [72], CHWs were named only three times, and emphasized as community representatives and as collaborators in health initiatives rather than respected health workers in need of support just as their clients are. CHWs are irreplaceable advocates for community well-being, and bolster the health of vulnerable communities; thus both research and policy should reflect their impact and value in order to truly advance health equity.

## Supporting information

S1 Fig Matrix displaying frequency of codes applied across qualitative dataset created via Dedoose. (TIFF)

S2 Fig Matrix displaying the presence or absence of codes applied across qualitative dataset created via Dedoose. (TIFF)

S3 Fig Matrix displaying the co-occurrence of codes applied across qualitative dataset created via Dedoose. (TIFF)

S1 Appendix Savoring Task Protocol. (DOCX)

S2 Appendix Study Summary and Findings Presented in Spanish. (DOCX)

S3 Appendix Representative Quotes within Each Identified Theme Translated from Spanish to English. (DOCX)
