# Supplementary material for: Savoring the service: A qualitative study of Latine community health workers’ health promotion efforts
Source: PLOS Glob Public Health. 2026 Aug 12;6(8):e0006654. doi: 10.1371/journal.pgph.0006654 (PMC13465823; doi:10.1371/journal.pgph.0006654)
Supplement: Supplementary file 1 [file pgph.0006654.s004.docx]

**S1 Appendix**

***Bolded text is intervener script. Note that, while the below protocol is in English for ease of review, the intervention was also (primarily) delivered in Spanish.**

*****Unbolded text is intervener instruction.

Savoring Task Protocol

**“Promotores/Community Services Providers** (say whichever term applies to the person) **often tell us they don’t have much time to focus on the positive experiences they’ve had helping the community because they are so busy doing their work and don’t have time to stop and reflect. And yet, we know that promotores/Community Services Providers are the “heart of the community,” lifting the community up and holding them together. You deserve a time to focus on all of the good that you are doing in your community and so the purpose of this program is to help you find time to focus on the positive change you are having in your corner of the world.**

**For the next few minutes, I am going to help you come up with a memory of a time when you felt extremely connected, close, helpful, or important in your community while you were doing your job as a promotora/Community Service Provider. I am especially interested in hearing about a time when you found joy in helping someone in your community reach new heights, or a time when someone needed you and you were there for them. It may be a time when you provided support for someone in your community who really needed you. Or it could be a time when you saw how the work you were doing lifted the whole community up. Feel free to choose something that you felt was a milestone or something simple that happens on a daily basis.**

**What I’d like you to do is try to come up with some memories and then I’ll help you decide which one to focus on for the next part of the study. Some people can think of just one and some people can think of several. It doesn’t really matter how many you can think of; we’re just going to be brainstorming. So can you tell me a time when you felt this way with your community or with a person in your community?”**

Ask the participant to tell you the memories that come to mind. After each one, ask the CSP how connected they felt to the person and the community and how detailed the memory is. Complete the Mental Reflection rating sheets as you discuss the memories with her. Keep in mind that we are really looking for memories of a time when the CSP acted as a secure base/safe haven for the community or for a member of the community (though you will not ask CSP specifically about this). You will then ask the CSP to focus on the memory that describes a time when they felt the most connected and can remember the most detail about the event. Remember, we are looking for positive memories. If possible, we would like them to choose a memory that is devoid of negative emotions. So a memory that contains both intensely positive and negative emotions will not be as good as a memory that contains only a less intense positive emotion. If the participant generates multiple memories of times when they felt extremely connected to the community with extensive detail, then you pick the one that has the strongest secure base/safe haven content. If the CSP generates multiple memories that are high on all of these qualities (positivity, detail, secure base/safe haven content), ask the participant which one she would like to focus on for this task.

When guiding the participant to the memory you’d like them to focus on, try to use a transition phrase that fits well for the situation. Some examples are:

- *It sounds like the first memory is* ***really salient in your mind****. Let’s focus on that one for today…*
- *Since the second memory was* ***more recent****, we’ll go with that one today…*
- *It sounds like* ***you have already spent some time processing*** *this memory so let’s go with the other one today…*

*If the participant does not generate any memories with explicit secure base/safe haven content (e.g., CSP comes up with three memories of activities she at work), then your job is to ask some additional questions to get the CSP into an attachment state of mind. Something like this:*

***It sounds like you really enjoyed being able to expose the person to something new or exciting. During that experience, did you notice moments when the person relied on you, or felt more comfortable because you were there? Were there things that he/she did that he/she wouldn’t have been able to do without the confidence of knowing you were there?***

*After you get the participant to that place, you will want to remind them of this attachment focus when directing them to focus on this memory:*

***Now, when focusing on this memory, I want you to really focus on the moments when your being there helped your community or a particular community member, and how you felt at those times.*** Tell the participant that you want them to focus on this memory while she completes this next task.

**“What’s going to happen now is that I’d like for you to sit comfortably and relax. I’m going to have you really focus on this memory for the next 10 minutes. I’ll ask you to focus on specific parts of the memory at different times and the feelings you had during this special moment with “the community/this person in your community.” You can feel free to relax and close your eyes if that will help you focus.**

**Now I’m going to ask you some questions and I’ll pause a bit in between so you have a chance to reflect on your feelings. For each question, I will give a few examples of things you may want to think about as you focus on this memory, but when you respond, don’t feel the need to answer each and every question. As you reflect on your feelings during that special moment with your community/this person in your community, please speak those feelings out loud. And if you run out of things to say, don’t worry about it and just keep thinking about the experience until I ask you another question. If you notice that your mind wanders to unrelated or negative thoughts, just gently bring it back to the positive aspects of this event. Does this sound okay? Do you have any questions?”**

*Record the stopwatch time before you begin reading the prompts.* Read the prompts slowly to the participants and give her plenty of time to reflect and respond.

1. **I’d like you to relax and begin focusing on the time when**	**with**

**the community/this person in your community***.* **First, I want you to notice and remember the details of the event. What was the weather like that day? What time of day did the event occur? What did**	*(other person/people)* **look like or what were they wearing? Do you remember what you were wearing? What kinds of sounds could you hear? What kinds of things could you see?**

2. **Now I’d like you to notice how you felt at this time. What kinds of things were you feeling in your body? Were you feeling happy and excited, or were you deeply calm and relaxed? Think about where in your body you felt these emotions and try to feel them now.**
3. **Now I’d like you to think about what you were thinking when**

*(memory).* **For example, were you thinking, “**	*(other person/people)* **really needs me at this moment and I’m there for them.**	**I’m so proud to have this role in my community. I am taking such good care of my community.” And what do you think about it now?**

4. **Now I’d like you to turn your focus to the future. Focus on how close you felt to**

*(other person/people)* **at that time. Focus on how close to felt to the broader community at that time. How will the bond that you have with the community now impact your ability to serve your community in the future? How will it affect your connection to the community? How will this bond affect the community overall? What positive things can you imagine happening as a result of your bond to one another?**

5. **For the last part of this reflection, please let your mind wander in any way you’d like related to this event. You may want to think about things I have asked you to think about earlier or you may want to think about how this memory is related to your other relationships and your life. It’s normal for your mind to wander to other topics or feelings – if you notice that your mind has wandered, just gently bring it back to the positive aspects of this event. Let your mind wander in any way you’d like, but try to keep focused on the positive, personal parts of this memory.**

Once ten minutes is up or when reflection has ended, say to participant:

**“Lastly, is there anything else you would like to say about this memory, or do you have any take-aways from engaging in this reflection process? Thank you so much for sharing this wonderful/important/meaningful memory with me. I look forward to meeting with you next week.”**
