# Supplementary material for: Savoring the service: A qualitative study of Latine community health workers’ health promotion efforts
Source: PLOS Glob Public Health. 2026 Aug 12;6(8):e0006654. doi: 10.1371/journal.pgph.0006654 (PMC13465823; doi:10.1371/journal.pgph.0006654)
Supplement: Supplementary file 2 [file pgph.0006654.s005.docx]

**S2 Appendix**

Los trabajadores de salud comunitarios (TSC) desempeñan un rol crucial en la salud de las comunidades latines de bajos recursos. Sin embargo, no existe suficiente información sobre cómo los TSC perciben las experiencias que tienen ayudando a la comunidad, y sobre cómo estas experiencias promueven su bienestar. Este estudio busca entender cómo los TSC se relacionan con las comunidades que sirven y cómo estas experiencias impactan su resiliencia. Los participantes de este estudio (N=30) trabajan en agencias comunitarias de salud que sirven a familias latines de bajos recursos en California. Los participantes completaron un proceso de reflexión guiado sobre momentos de servicio a la comunidad y respondieron una batería de preguntas sobre agotamiento laboral y psicopatología. Los análisis cualitativos demostraron que los TSC actúan como una red de apoyo, proporcionando recursos, cultivando la independencia de sus clientes, y apoyándolos inclusive haciendo más de lo requerido por su trabajo. Así mismo, los TSC perciben experiencias satisfactorias en su trabajo, incluyendo sentimientos de satisfacción sobre los logros de sus clientes o la colaboración con compañeros de trabajo, y percepción de beneficios propios (emocionales o monetarios). No obstante, los TSC también perciben desafíos, los cuales incluyen sentimientos de fatiga emocional y ocupacional, falta de valoración externa, y frustración ante barreras sistémicas. Finalmente, los participantes describieron fuentes de resiliencia -expresiones de gratitud por parte de los clientes, conexión con la comunidad, motivación intrínseca de ayudar a otros, espiritualidad o fe- que contribuyen a su fortaleza emocional. Estrategias como programas de apoyo entre colegas, enfoques personalizados para fomentar resiliencia, y reconocimiento del impacto del trabajo pueden promover la resiliencia en los TSC. Aun así, el apoyo a nivel sistémico, incluyendo compensación monetaria y consideraciones políticas, es necesario para fomentar el bienestar de los TSC.
