# Supplementary material for: Savoring the service: A qualitative study of Latine community health workers’ health promotion efforts
Source: PLOS Glob Public Health. 2026 Aug 12;6(8):e0006654. doi: 10.1371/journal.pgph.0006654 (PMC13465823; doi:10.1371/journal.pgph.0006654)
Supplement: Supplementary file 3 [file pgph.0006654.s006.docx]

**S3 Appendix**

*Representative Quotes within Each Identified Theme Translated from Spanish to English*

Support Net

(*Indirect Impact, Direct Impact, Applying Unique Skills and Experiences, Going Above and Beyond*)

- *“I feel responsible that if I have, for example, my abilities to make crafts I feel responsible that I have to teach people...and I feel I feel happy that I can teach others my abilities, and that those people can then teach [others].”*
- *“And this is for me more than me helping fiscally-that is, directly to a person, for me I feel that I am not helping but leaving something planted mhm right? And something sown is that that person can be independent and not have to come all the time to the promoter [Name] or the promoter [Name] or whoever to be able to resolve their, their issues…”*
- *“Everything was like good for me, it was good like that, very gratifying, because I felt good, useful because you helped a lot of people… But gratifying because you helped a lot of people with–with food and with this financial help. There was–there was a–there was a help. And–and they helped each other with their clients so that they could open, so that they could eat. It went well and I liked it. I felt very useful at that moment.”*
- *“Ah uh, since she speaks pure English, the one we are in, the one I put in school was her husband…I sent him because he didn’t want to and I told him, ‘Yes, look you have to do it’ and he said, ‘No’ that he will not want to do it. I told him, ‘No, yes, you are going to do it because I am telling you to.’ And so I enrolled him, I sent him and I am very proud because he is very consistent in his classes. He is on time to class, does his homework, I mean, he is taking it like really, really, something like really valuable to him.”*

Fulfilling Experiences

(*Satisfaction with Community Health Work, Community Connections, Personal Benefit, Gratitude from Clients*)

- *“Well, it is--it is-- personally I do have that satisfaction of-of saying or maybe I have helped a bit, but I have contributed a bit to my community and also that I have realized that- for example, our community is a community that needs a lot of help… I think I can contribute so that the resources arrive to them, for them to take advantage of the resources that exist, because when I say that there are resources for us to take advantage of and for these to be utilized by those who really need it, right? That to me--me. Well, yes, it creates a lot of satisfaction and at the same time like suddenly to live, I think.”*
- *“Mmm, well generally uh we feel very supported. The truth is, in this country, in this city, I am the only one from my family with my son, so practically they are my family, all my memories are with them.”*
- “*I think that it is super good because every person that comes into my life impacts me in some way, but at the same time I impact their lives. It’s like-like a mutual learning [process] and the support is mutual, because when I lost my mom, they were with me, they brought me flowers, umm they cooked for me so that I wouldn’t have to cook. Umm, when they evicted me they would tell me, ‘This is your house, don’t worry. Come here to the--to our house’s yard. We will set up a tent. You are not alone.’*”
- *“[My client said] ‘I attended the [event] and thanks to the [services] that you did with us, you gave me the desire to live, I changed, I changed my way of thinking.' And she thanked me very much, so that left me totally impacted like, there are others memories, right, of other projects that I'm also involved in, [smacks lips] but this was the one that left me the most impacted, and of-- of satisfaction and knowing that perhaps those two hours that we met a week we were working on--on her without realizing it and to change that way of thinking..."*

Challenges of Community Health Work

(*Sadness, Burnout, Helplessness, Overwhelm, Stress, Frustration, Worry*)

- *“If they got sick, they died because sometimes they arrived at the clinics very, the people, very unwell, very unwell, I mean with those fevers and almost passing out and [they] would arrive at the clinics infected people and a lot of people. Even though it was painful because a lot of people started to lose their family members.”*
- *“To the point where we can put our limits ourselves, because we have to take care of our integrity, right? We cannot risk our lives for other people. And even more if [they] don’t want to receive help. So yeah…until now I don’t see that-that we play an important role or that-that our job is totally recognized because it is not recognized.”*
- *“Sometimes, you know, when you feel like, okay, you’re maybe not doing enough for someone and you kind of feel like I couldn’t help them maybe mhm….Like, I just there was no help for this person. So I had to just leave them in the middle of mhm their situation.”*
- *“I imagined economically [they] will use a lot of help and that was more like my-my worry if you can say that, because I said, how will we help this family?...it will need we will need a lot of help with this family economically and more, not-not economically, emotionally too, but also because I saw that they were it looked like they were very humble, economically like not—not too stable.”*
- *“Sometimes it’s a little, we can talk with a lot of people to help [them] but really what [you] are doing is how we feel at the same time we are helping and sometimes we ask ourselves mhm ‘how do I feel?’ Because these people don’t ask me how do I feel. Maybe I have problems, I have to bring out [my] joy to the people so they don’t see me sad, nor frustrated, or sometimes I have depression problems but I have to face the community because the community too, well [they] go through worse things that oneself.”*
- *“I’m very critical…not satisfied nor proud mhm I will feel proud when, when less youth are criminalized, [when] I see that the system is starting to do something for the youth.”*
- *“Well, when um—when I started [Service] I thought ‘What will I teach them, no? What will I tell them? What will I talk about? Um, what topics do I have to study? Mostly I thought like um like more like arriving and teaching like a class and that [they] write [things] down, no?... so I had those those thoughts of doubt, no? Of—of fear that I will not be able to.”*

Sources of Resilience

(*Gratitude from Clients, Inner Drive, Relatable Experiences, Spirituality*)

- *“Well yes I felt happy like no, no, I don’t want to cry because it tickled my stomach of happiness and of surprise. And it’s me and happiness. Happiness that someone came to my door and brought me flowers…I was all happy and with everyone that would want to talk to me I would tell them ‘this happened to me’ and that day I would tell them the story, but yes I go [feeling] well, I liked it.”*
- *“Well you see, in that moment my goal was to fulfill the need of the lady, which was the vaccine, mhm mhm, so I focused on that to see in what way I could help her, how I could help her, because I did not want to let her down, right right, I didn’t want her to feel like--like--feel insecure with me and later I be like ‘nothing can be done’, no no no, I was very focused in being able to satisfy her need.”*
- *“Well this is a success for us, because we were there at some moment in our lives, and I think that is the success of promotoras. The ‘oh shoot, me too, I can act and help.’ And you do it with all your heart. Of course. It’s the experience, then, that you understand the interconnectedness because of the [shared] experience…”*
- *“...I always try to always give thanks to God because I say ‘Yes, wow, I don’t know how it happened, but I did it and thanks to God that gave me the opportunity to do it’, right? Because I always thought of saying ‘Father, I have this and that type of responsibility that I carry, but without your help I will not be able to do it’ and I’m praying that everything goes well.”*
