# Supplementary material for: Savoring the service: A qualitative study of Latine community health workers’ health promotion efforts
Source: PLOS Glob Public Health. 2026 Aug 12;6(8):e0006654. doi: 10.1371/journal.pgph.0006654 (PMC13465823; doi:10.1371/journal.pgph.0006654)
Supplement: Supplementary file 4 [file pgph.0006654.s007.docx]

Overall, I enjoyed reading the article. It is coherently written and fluently presented. The main contribution of this research lies in its use of a novel technique—relational savour­ing (RS)—as a research tool to explore how CHWs engage with their client communities and derive meaning from these experiences. Another key contribution is that, although the study uses secondary data, it employs multiple techniques (e.g., member checking, debriefing) to enhance the quality and trustworthiness of its findings. The authors identify four primary themes: Fulfilling Experiences, Sources of Resilience, Challenges of Community Health Work, and Support Net.

While the study deliberately focuses on the positive experiences of CHWs, it also highlights systemic and structural barriers they face, including inadequate compensation and limited recognition of their work. These findings are relevant and align with global literature on CHWs.

I have several suggestions to strengthen the manuscript, mainly regarding the findings, discussion, and conclusion sections. In some places, I could not be specific because the line and page numbers disappear partway through the document. I have *italicised the text taken directly from the manuscript* to help the authors identify areas for revision.

**Specific Comments**

**Line 240**
How long was spent on Part 1 of the RS exercise? Was there a time limit for participants to recall and describe a memory?

**Lines 270–271**
The manuscript states: *“We additionally employed several methods to establish trustworthiness, maximizing accountability, replicability, and rigor.”*
However, it is unclear how these principles were operationalized in this specific study. Instead of providing broad theoretical definitions, please demonstrate concretely how each principle was applied.

**Line 339**
You refer to the code frequency matrix, the code presence matrix, and the code co-occurrence matrix. If it is possible, it may be useful to include screenshots of these as supplementary material so readers can better understand what was analysed.

**Table 2: Representative Quotes (Translated from Spanish to English)**

Although the table provides relevant quotes for each theme, it is difficult to interpret them without accompanying descriptions of the themes themselves. It may be more effective to move this table to the appendix and incorporate selected quotes directly into the narrative of each research question. This will also reduce duplication, as some quotes appear both in the table and earlier sections.

**Research Questions**

**RQ1a – How do Community Health Workers Engage with the Community?**
While one quote is provided at the end of this section, it would strengthen the analysis to outline each key way CHWs engage with the community and pair each with a supporting quote.

**RQ1b – What Service Experiences Are Particularly Salient and Impactful?**
The manuscript emphasises that CHWs commonly described benefits and positive effects of their roles. Since the study intentionally focused on positive experiences, it would be helpful to acknowledge explicitly that participants were encouraged to reflect on positive memories. Despite this, CHWs raised challenges—highlighting the emotionally demanding nature of community work, often performed by women.

The manuscript notes that *“Some CHWs expressed frustration with and anger around systemic barriers or challenges that the broader community was facing.”*
It would strengthen this claim to include a representative quote.

**Discussion**

The manuscript states that the study used a *“mixed-methods approach”*. This term is not explained in the Methods section. If “mixed-methods” refers to the use of RS combined with qualitative thematic analysis, this should be clearly articulated in the Methodology.

A large portion of the Discussion is not sufficiently engaged with the wider global literature on CHWs. For example, the manuscript states that CHWs require emotional and financial support systems, but does not cite research to support this argument. It would be valuable to include evidence from international contexts that emphasise the importance of adequate remuneration and support for CHWs. While U.S.-based literature is understandably prominent, acknowledging global perspectives would strengthen the contribution and situate the findings within broader debates.

Similarly, the suggestion to introduce peer-support models for CHWs should be supported with evidence demonstrating the effectiveness of such approaches.

The manuscript correctly identifies systemic and structural barriers faced by CHWs. However, some of the proposed solutions—such as expanding mental health support—do not fully address the structural issues identified. For instance, CHWs reported needing fair pay and formal recognition. These aspects should be incorporated more directly into the recommendations.

**Certification**

The issue of certification appears in the Discussion but is not presented in the Findings. I recommend including participants’ perspectives on certification in the Results section, supported by quotes where relevant, and then discussing this issue in relation to the broader literature.

**Conclusion**

The Conclusion highlights the issue of CHW certification, but since this has not been presented in the Results, the argument feels disconnected. Please consider moving the findings related to certification from the Discussion into the Results and then revisiting this topic in the Conclusion.
