# Supplementary material for: Savoring the service: A qualitative study of Latine community health workers’ health promotion efforts
Source: PLOS Glob Public Health. 2026 Aug 12;6(8):e0006654. doi: 10.1371/journal.pgph.0006654 (PMC13465823; doi:10.1371/journal.pgph.0006654)
Supplement: Supplementary file 5 [file pgph.0006654.s009.docx]

Dear Dr. Dhaliwal,

We appreciate your invitation to revise and resubmit our manuscript entitled, "Savoring the service: A qualitative study of Latine community health workers’ health promotion efforts" for review to *PLOS Global Public Health*. We thank the editorial team and reviewers for their thoughtful comments and have made the suggested revisions, which have resulted in an improved manuscript. This letter details our point-by-point responses to the comments provided by the reviewers, who each offered valuable critiques of our manuscript. In the table below, we quote reviewers’ comments, note the relevant section of the paper, provide our response, and indicate the relevant page number of the manuscript where revisions can be found. Please note that we have also changed the corresponding author for the manuscript.

It is our hope that we have adequately addressed the recommendations offered to us, and we look forward to hearing from you.

| **Source of Feedback** | **Section of Paper** | **Editor/Reviewer Comments** | **Authors’ Response** | **Page** |
| --- | --- | --- | --- | --- |
| **Journal** | All | Please amend your detailed Financial Disclosure statement. This is published with the article. It must therefore be completed in full sentences and contain the exact wording you wish to be published.  i. Please clarify all sources of financial support for your study. List the grants, grant numbers, and organizations that funded your study, including funding received from your institution. Please note that suppliers of material support, including research materials, should be recognized in the Acknowledgements section rather than in the Financial Disclosure.  ii. State the initials, alongside each funding source, of each author to receive each grant. For example: "This work was supported by the National Institutes of Health (####### to AM; ###### to CJ) and the National Science Foundation (###### to AM)." iii. State what role the funders took in the study. If the funders had no role in your study, please state: “The funders had no role in study design, data collection and analysis, decision to publish, or preparation of the manuscript.” iv. If any authors received a salary from any of your funders, please state which authors and which funders. | This change has been made. | p. 33 |
|  | All | In the online submission form, you indicated that “The datasets analysed during the current study are not publicly available due to the qualitative nature of the data itself and cannot be made available on request–that is, because qualitative data inherently includes rich and narrative information, it is impossible to fully protect participant confidentiality and release raw qualitative data even if explicit identifying information is removed.”.  All PLOS journals now require all data underlying the findings described in their manuscript to be freely available to other researchers, either  1. In a public repository,  2. Within the manuscript itself, or  3. Uploaded as supplementary information.  This policy applies to all data except where public deposition would breach compliance with the protocol approved by your research ethics board. If your data cannot be made publicly available for ethical or legal reasons (e.g., public availability would compromise patient privacy), please explain your reasons by return email and your exemption request will be escalated to the editor for approval. Your exemption request will be handled independently and will not hold up the peer review process, but will need to be resolved should your manuscript be accepted for publication. One of the Editorial team will then be in touch if there are any issues. | Thank you for this request. While we understand and acknowledge the importance of data availability, we will not be able to share our sensitive qualitative data for this particular study for multiple reasons. First, qualitative data is much more identifiable than quantitative data, even with identifying information removed. Second, our sample is very small and primarily recruited from a single community service agency, exacerbating identifiability concerns. Third, our sample is a vulnerable sample according to our community partner, who recommended against broader data sharing to reduce risk to participants, many of whom are undocumented. | — |
|  | Methods | 3. Please ensure that your Ethics Statement is available in its entirety at the beginning of your Methods section, under a subheading 'Ethics Statement'. It must include:  i) The name(s) of the Institutional Review Board(s) or Ethics Committee(s)  ii) The approval number(s), or a statement that approval was granted by the named board(s)  iii) (for human participants or donors) - A statement that formal consent was obtained (must state whether verbal/written) OR the reason consent was not obtained (e.g., anonymity).  vi) (for child participants) - Please update your Ethics Statement with confirmation that written informed consent was obtained from the parent/guardian of each participant under 18 years of age. | This change has been made. | Pp. 11 |
|  | Supplementary | 4. Please provide separate figure files in .tif or .eps format.  For more information about figure files please see our guidelines:  [https://journals.plos.org/globalpublichealth/s/figures](https://urldefense.com/v3/__https://journals.plos.org/globalpublichealth/s/figures__;!!LIr3w8kk_Xxm!ufg6ETNcqiDPaqfAKL3j6l1uAGPogX27eZrAScq6XdkRpGnR_Iryna6e8zsS0Gdx1cMPu4q4AnFIs2yQ$)  [https://journals.plos.org/globalpublichealth/s/figures#loc-file-requirements](https://urldefense.com/v3/__https://journals.plos.org/globalpublichealth/s/figures*loc-file-requirements__;Iw!!LIr3w8kk_Xxm!ufg6ETNcqiDPaqfAKL3j6l1uAGPogX27eZrAScq6XdkRpGnR_Iryna6e8zsS0Gdx1cMPu4q4AmanbSbm$) | This change has been made. | — |
|  | Supplementary | We have noticed that you have uploaded Supporting Information files, but you have not included a list of legends. Please add a full list of legends for your Supporting Information files after the references list.  If the reviewer comments include a recommendation to cite specific previously published works, please review and evaluate these publications to determine whether they are relevant and should be cited. There is no requirement to cite these works unless the editor has indicated otherwise.   Please review your reference list to ensure that it is complete and correct. If you have cited papers that have been retracted, please include the rationale for doing so in the manuscript text, or remove these references and replace them with relevant current references. Any changes to the reference list should be mentioned in the rebuttal letter that accompanies your revised manuscript. If you need to cite a retracted article, indicate the article’s retracted status in the References list and also include a citation and full reference for the retraction notice. | This change has been made. We believe there is only one legend (for Figure 1) that was needed. We have included this as a separate file. | — |
| **Reviewer 1** | All | Well written manuscript with a detailed explanation of methodology which was easy to follow | We are grateful for the Reviewer’s helpful feedback throughout this process! | — |
|  | Introduction | Add some statistics on the number of CHWs in California / US; criteria for recruitment; their roles and responsibilities; work hours; wages - in the Introduction | Thank you for this suggestion. We have added relevant statistics regarding professional demographics of CHWs in California and in the broader US in the Introduction. | pp. 5 |
|  | Introduction | Look for published research on the mental health and work stress of CHWs - specifically prevalence of anxiety, stress and burnout | We thank you for this recommendation. We have incorporated additional research on the mental health of CHWs. | pp. 7 |
|  | Methods | Was there a ‘number of working years’ in the inclusion criteria? | We thank the Reviewer for this question. There was not a ‘number of working years’ requirement in the inclusion criteria. The only inclusion criteria was that participants were at least 18 years of age and work as a community service provider at a community service agency. The average number of years that our participants have worked as a community service provider was 9.33 years (*SD* = 7.82 years). | — |
|  | Discussion | Suggestion: to look into studies done on CHWs in India (especially the Accredited Social Health Activists - ASHAs) and other South East Asian countries for comparisons in your discussion (even though the demography and study settings are different, the themes generated in this study can be compared) | We thank you for this suggestion. We have incorporated studies conducted in India for comparison in the Discussion. | pp. 35-36 |
| **Reviewer 2** | All | I enjoyed reading the article. It is coherently written and fluently presented. The main contribution of this research lies in its use of a novel technique—relational savour¬ing (RS)—as a research tool to explore how CHWs engage with their client communities and derive meaning from these experiences. Another key contribution is that, although the study uses secondary data, it employs multiple techniques (e.g., member checking, debriefing) to enhance the quality and trustworthiness of its findings. The authors identify four primary themes: Fulfilling Experiences, Sources of Resilience, Challenges of Community Health Work, and Support Net. While the study deliberately focuses on the positive experiences of CHWs, it also highlights systemic and structural barriers they face, including inadequate compensation and limited recognition of their work. These findings are relevant and align with global literature on CHWs. I have several suggestions to strengthen the manuscript, mainly regarding the findings, discussion, and conclusion sections, which is attached separately. | We are grateful for the Reviewer’s helpful feedback throughout this process! | N/A |
|  | All | In some places, I could not be specific because the line and page numbers disappear partway through the document. I have *italicised the text taken directly from the manuscript* to help the authors identify areas for revision. | We thank the effort that the Reviewer has made to provide clear feedback. We have addressed the missing line and page numbers. | — |
|  | Methods | **Line 240**  How long was spent on Part 1 of the RS exercise? Was there a time limit for participants to recall and describe a memory? | Thank you for this question. There was not a standard length of time spent on Part 1 of the RS exercise. There was no time limit for participants to recall and describe a memory. Still, session lengths were typically around 30 minutes, including both parts of the RS exercise. | — |
|  | Methods | **Lines 270–271** The manuscript states: *“We additionally employed several methods to establish trustworthiness, maximizing accountability, replicability, and rigor.”* However, it is unclear how these principles were operationalized in this specific study. Instead of providing broad theoretical definitions, please demonstrate concretely how each principle was applied. | We thank you for this recommendation. We have incorporated specific examples of how each principle was concretely applied in this specific study. | pp. 16 |
|  | Methods | **Line 339** You refer to the code frequency matrix, the code presence matrix, and the code co-occurrence matrix. If it is possible, it may be useful to include screenshots of these as supplementary material so readers can better understand what was analysed. | We thank you for this comment. We have included screenshots of each matrix in the supplementary material. | — |
|  | Results | **Table 2: Representative Quotes (Translated from Spanish to English)**  Although the table provides relevant quotes for each theme, it is difficult to interpret them without accompanying descriptions of the themes themselves. It may be more effective to move this table to the appendix and incorporate selected quotes directly into the narrative of each research question. This will also reduce duplication, as some quotes appear both in the table and earlier sections. | We thank you for this recommendation. We have moved the table to the appendix and incorporated selected quotes directly into the narrative of each research question to reduce duplication. | — |
|  | Results | **RQ1a – How do Community Health Workers Engage with the Community?** While one quote is provided at the end of this section, it would strengthen the analysis to outline each key way CHWs engage with the community and pair each with a supporting quote. | We thank you for this suggestion. We have incorporated additional quotes to outline each key way that CHWs engage with the community. | — |
|  | Results | **RQ1b – What Service Experiences Are Particularly Salient and Impactful?** The manuscript emphasises that CHWs commonly describe benefits and positive effects of their roles. Since the study intentionally focused on positive experiences, it would be helpful to acknowledge explicitly that participants were encouraged to reflect on positive memories. Despite this, CHWs raised challenges—highlighting the emotionally demanding nature of community work, often performed by women. | We thank you for this feedback and agree that it would be important to emphasize that CHWs raised challenges even when prompted to recall positive memories. We have revised as such. | pp. 25 |
|  | Results | The manuscript notes that *“Some CHWs expressed frustration with and anger around systemic barriers or challenges that the broader community was facing.”* It would strengthen this claim to include a representative quote. | We thank the Reviewer for this suggestion and agree that a representative quote would strengthen this claim. We have included two representative quotes. | pp. 28 |
|  | Discussion | The manuscript states that the study used a *“mixed-methods approach”*. This term is not explained in the Methods section. If “mixed-methods” refers to the use of RS combined with qualitative thematic analysis, this should be clearly articulated in the Methodology. | Thank you for this question. We have removed this language to minimize confusion. We intended to convey that this study was part of a larger trial that included quantitative and qualitative approaches. However, the current study uses only qualitative analysis. | — |
|  | Discussion | A large portion of the Discussion is not sufficiently engaged with the wider global literature on CHWs. For example, the manuscript states that CHWs require emotional and financial support systems, but does not cite research to support this argument. It would be valuable to include evidence from international contexts that emphasise the importance of adequate remuneration and support for CHWs. While U.S.-based literature is understandably prominent, acknowledging global perspectives would strengthen the contribution and situate the findings within broader debates. | We agree with the Reviewer that incorporating more global research on CHWs would greatly strengthen this manuscript. We have now included such literature in the Discussion section. | pp. 35 |
|  | Discussion | Similarly, the suggestion to introduce peer-support models for CHWs should be supported with evidence demonstrating the effectiveness of such approaches. | We thank you for this suggestion. We have added additional information about this recommendation and a scoping review demonstrating the benefits of such models in healthcare settings. | pp. 31 |
|  | Discussion | The manuscript correctly identifies systemic and structural barriers faced by CHWs. However, some of the proposed solutions—such as expanding mental health support—do not fully address the structural issues identified. For instance, CHWs reported needing fair pay and formal recognition. These aspects should be incorporated more directly into the recommendations. | We thank the Reviewer for this important point. We have more explicitly and directly stated these recommendations around fair pay and formal recognition into the Discussion. | pp. 35 |
|  | Results, Discussion | The issue of certification appears in the Discussion but is not presented in the Findings. I recommend including participants’ perspectives on certification in the Results section, supported by quotes where relevant, and then discussing this issue in relation to the broader literature. | We thank you for this suggestion. We have moved the findings related to certification from the Discussion into the Results. We have also added additional reflections on the issue of certification into the Conclusion. Please note that we did not include participant quotes because these findings were noted as part of focus groups intended to be used to check our assumptions and findings against what the participants believed. Thus, broad takeaways and recommendations from the discussion were noted to be included in the manuscript, but the focus group itself was not recorded in any way and specific quotes are not available to be included. | pp. 28 |
|  | Results, Discussion | The Conclusion highlights the issue of CHW certification, but since this has not been presented in the Results, the argument feels disconnected. Please consider moving the findings related to certification from the Discussion into the Results and then revisiting this topic in the Conclusion. | We thank you for this suggestion. We have moved the findings related to certification from the Discussion into the Results. | pp. 28 |
